# Nutritional epigenomic and DNA-damage modulation effect of natural stilbenoids

**DOI:** 10.1038/s41598-022-27260-1

**Published:** 2023-01-12

**Authors:** Sara Volpes, Ilenia Cruciata, Federica Ceraulo, Chiara Schimmenti, Flores Naselli, Cecilia Pinna, Maurizio Mauro, Pasquale Picone, Sabrina Dallavalle, Domenico Nuzzo, Andrea Pinto, Fabio Caradonna

**Affiliations:** 1 Dipartimento di Scienze e Tecnologie Biologiche Chimiche e Farmaceutiche, Università di Palermo, (STEBICEF - Sezione di Biologia Cellulare), Viale delle Scienze, Edificio 16, 90128 Palermo, Italy; 2 Dipartimento di Scienze per gli Alimenti, la Nutrizione e l’Ambiente, DeFENS, Università degli Studi di Milano, Via Celoria 2, 20133 Milano, Italy; 3 Department of Obstetrics & Gynecology and Women’s Health, Albert Einstein College of Medicine, Michael F. Price Center 1301 Morris Park Avenue, Bronx, NY 10461 USA; 4 Istituto per la Ricerca e l’Innovazione Biomedica (IRIB), Consiglio Nazionale delle Ricerche (CNR), Via Ugo la Malfa, 153, 90146 Palermo, Italy

**Keywords:** Epigenetics, DNA methylation, Biochemistry, DNA, Chromosomes, Genetics, Cancer epigenetics, Nutrigenomics

## Abstract

The aim of the present work is the evaluation of biological effects of natural stilbenoids found in *Vitis vinifera*, with a focus on their activity as epigenetic modulators. In the present study, resveratrol, pterostilbene and for the first time their dimers (±)-*trans*-δ-viniferin, (±)-trans-pterostilbene dehydrodimer were evaluated in Caco-2 and HepG-2 cell lines as potential epigenetic modulators. Stilbenoids were added in a Caco-2 cell culture as a model of the intestinal epithelial barrier and in the HepG-2 as a model of hepatic environment, to verify their dose-dependent toxicity, ability to interact with DNA, and epigenomic action. Resveratrol, pterostilbene, and (±)-trans-pterostilbene dehydrodimer were found to have no toxic effects at tested concentration and were effective in reversing arsenic damage in Caco-2 cell lines. (±)-*trans*-δ-viniferin showed epigenomic activity, but further studies are needed to clarify its mode of action.

## Introduction

Health benefits associated with Mediterranean diet are correlated to the significantly large intake of fruits, vegetables, cereals, legumes, nuts, wine, beer, and olive oil, containing a plethora of bioactive molecules, especially phenolic compounds, which possess important biological properties such as anticancer, anti-inflammatory, and antioxidant and neuroprotection activities^1–5^.

Recent studies have evidenced the potential of polyphenols as anticancer compounds, mainly acting through epigenetic mechanisms^6,7^.

Epigenome irregularities, like DNA hypomethylation and hypermethylation, modulation of particular miRNA and histone deacetylation are indicated as markers in cancerous cells^8^. Food components, minerals and vitamins can be involved in epigenetic/epigenomic changes in cells through mechanisms that are not completely understood yet. Nutrigenomics studies the nutrients-genome interactions and in particular the effects of nutrients on DNA methylation pattern, histone modifications and miRNA action^9^.

Epigenetic modifications can play a significant role in disease occurrence and pathogenesis including cancer, cardiometabolic alterations and neurological diseases^9–11^. The changes in epigenetics are slow and progressive but can be possibly reversed by certain nutrients involved in histone modifications and/or methylation process of DNA^12^.

Stilbenoids are a group of natural phenolic compounds which have been isolated from various plant species, including grapes, peanuts, cranberries, and other botanical sources^13^.

Ranging from monomers to octamers, stilbenoids form one of the most interesting and therapeutically relevant groups of plant-derived polyphenols. Stilbenoids, like pterostilbene and resveratrol, have many proprieties, e.g., pterostilbene can induce genetic changes related to apoptosis-like cell death, such as an increase and decrease in, respectively, REC-A and LEX-A gene expression involved in antibacterial activity^14^. Both resveratrol and pterostilbene have effect in Central Nervous System: it was demonstrated that resveratrol exerts neuroprotective effects on central features of Alzheimer's disease and pterostilbene appears to be more effective in combatting brain changes associated with aging^15^. Moreover, (±)-*trans*-δ-viniferin and (±)-trans-pterostilbene dehydrodimer, have been studied in view of the interaction with duplex and G-quadruplex DNA^6^. By considering all these preliminary data, it seemed interesting and reasonable to hypothesize that these molecules can act at epigenetic/epigenomic level.

In the present work two monomers, resveratrol and pterostilbene, and their dimers (±)-*trans*-δ-viniferin and (±)-trans-pterostilbene dehydrodimer, were selected and evaluated in Caco-2 and HepG-2 cell lines as potential epigenetic modulators with the aim to reveal their epigenetic effects both in an in vitro cell model of normal human gut and in a tumoral hepatic cell line. Tested concentrations were chosen to mimic actual concentrations in gut lumen and liver^16^. Moreover, only in Caco-2 cell line as an in vitro model of normal human intestinal epithelium, the epigenomic recovery property of a selected stilbenoid after an (epi)mutagenic pre-treatment with a natural pollutant contained in drinking water, like arsenic, was assessed.

## Results

### Morphologically observations

**Figure 1:**
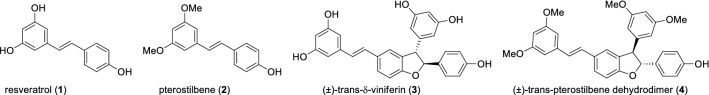
Structures of the investigated stilbenoids. For easier identification, the stilbenoids are numbered (see text for explanation).

Morphological observations were carried out to evaluate whether treatment with selected stilbenoids caused cellular changes. We did not find significant changes in cell morphology after the treatment with stilbenoids **1**, **2** and **4** (Fig. 1).

Stilbenoid **3**, (±)-*trans*-δ-viniferin, at tested concentration, showed cytotoxic effects.

#### (±)-*trans*-δ-viniferin (stilbenoid 3, Fig. 1) morphologically modifies differentiated Caco-2 cells

**Figure 2:**
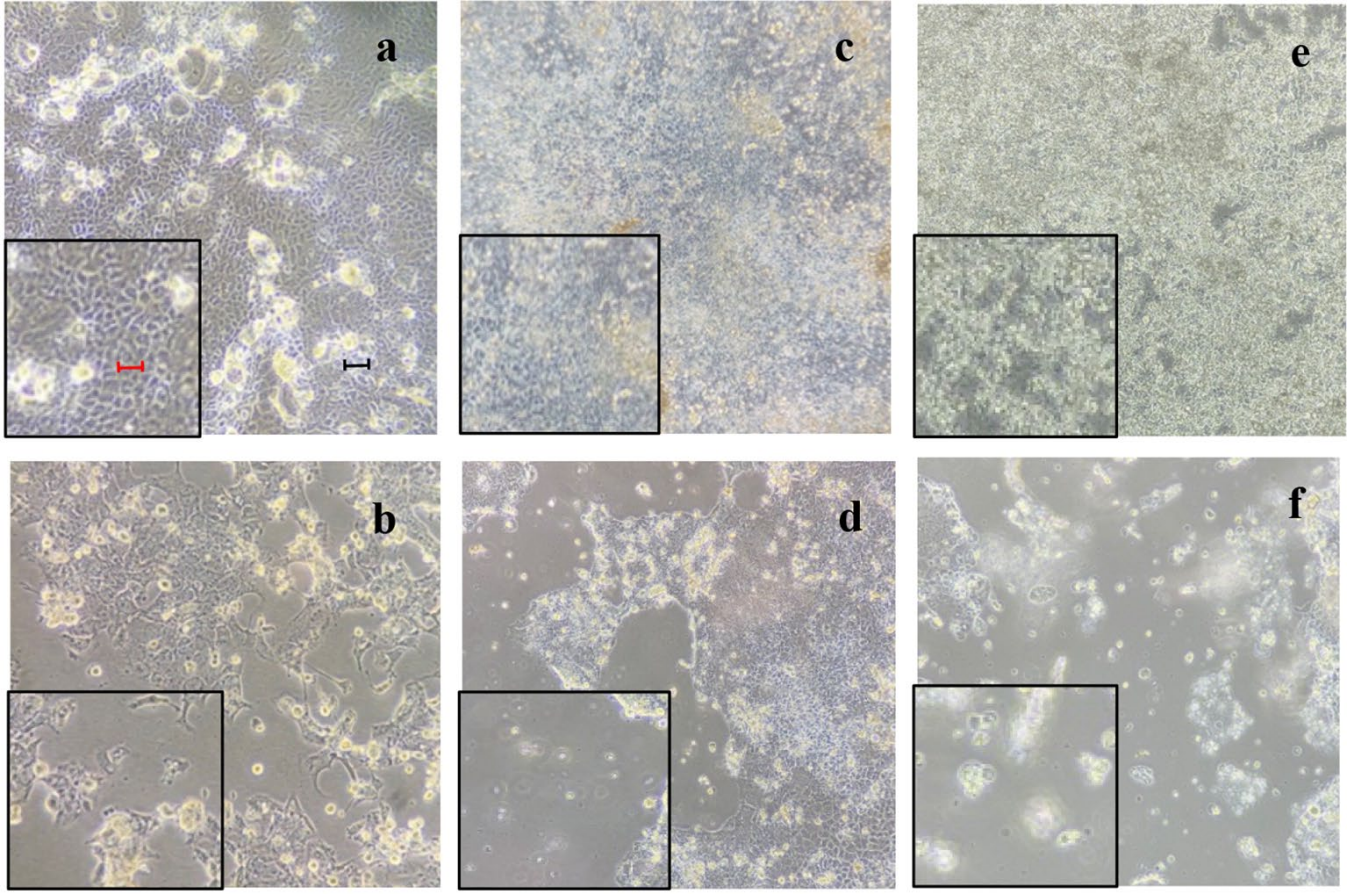
Microscopic observations of differentiated Caco-2 cell morphology after treatment with: (**a**) DMSO vehicle only, the cells are all well adherent to the substrate and appear whit a physiological morphology; (**b**) 5-AzaC positive control, is evident an inhomogeneous cellular mat and contracted cells; (**c**) VF20, some cells appear detached and rounded (evident in the difficulty of focusing); (**d**) VF200, an evident inhomogeneous cellular mat; (**e**) B33, cells still adhering to the substrate appear however rounded; (**f**) B75, most of the cells lose adhesion to the substrate, also evident in the difficulty of focusing. Every picture reports a 5 × magnification on the left for a better image evaluation. Black bar represents 10 µm, the red one, 50 µm.

The specific microscope fields are shown in Fig. 2. Compared to culture with DMSO (Fig. 2a), treatment with both concentrations of (±)-*trans*-δ-viniferin (VF20 and VF200–Fig. 2c, d) showed suffering cells (i.e. cells did not maintain their morphological conditions in culture and appeared shriveled and detached from the substrate); this was most evident in VF200. Moreover, there was a difference in morphology and in cell density after the treatment with 5-AzaC (Fig. 2b) since cells appeared distressed and contracted. The treatment with the two concentrations of B339H (Fig. 2e, f) caused the loss of adhesion to the substrate, mainly at the maximum concentration, whereas at minimum concentration cells still adhering to the substrate appeared however suffering.

#### (±)-*trans*-δ-viniferin concentration morphologically modifies HepG-2 cells

**Figure 3:**
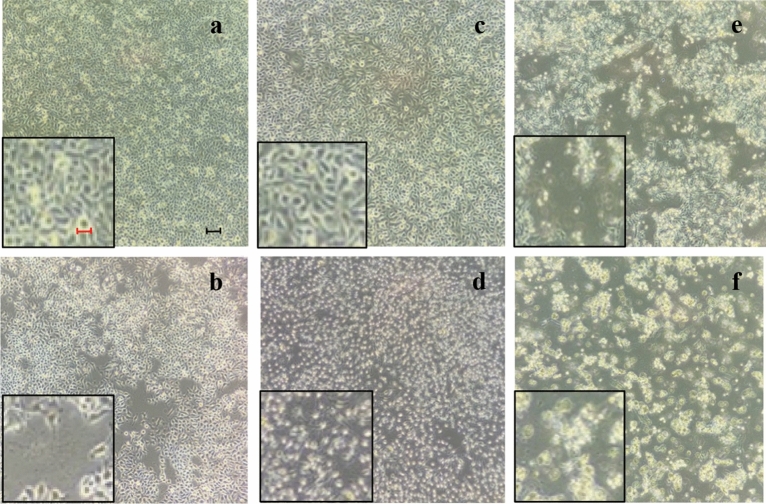
Microscopic observations of HepG-2 cell morphology after 72 h from treatment with: (**a**) DMSO vehicle only, the cells are all well attached and appear with their physiological morphology; (**b**) 5-AzaC positive control, cells still adhering to the substrate appear however bubbled and partially detached; (**c**) VF20, cells appear similar to cells with DMSO only; (**d**) VF200, cells appear contracted; (**e**) B33, cells still adhering to the substrate appear suffering and many cells lose adhesion to the substrate; (**f**) B75 treatment induce the detachment of cells from the substrate. Every picture reports a 5 × magnification on the left for a better image evaluation. Black bar represents 10 µm, the red one, 50 µm.

The HepG-2 cells treated with (±)-*trans*-δ-viniferin and the two control molecules (5-AzaC, B33, B75) showed morphological changes (Fig. 3).

Compared to the culture with only DMSO- vehicle (Fig. 3a), the treatment with (±)-*trans*-δ-viniferin (Fig. 3c, d) induced a cell morphological variation at the 200 µM where the cells appeared suffering and contracted.

A difference in morphology and in cell density was also observed following treatment with 5-AzaC (Fig. 3b), compared to DMSO vehicle (Fig. 3a). In fact, there was an inhomogeneous cellular mat and cells particularly contracted and suffering.

The treatment with the two concentrations of B339H (Fig. 3e, f) caused the loss of adhesion to the substrate, evident from the loss of focus under the microscope.

#### Treatment with stilbenoids is able to recovery sodium arsenite-induced damage in Caco-2 cells

**Figure 4:**
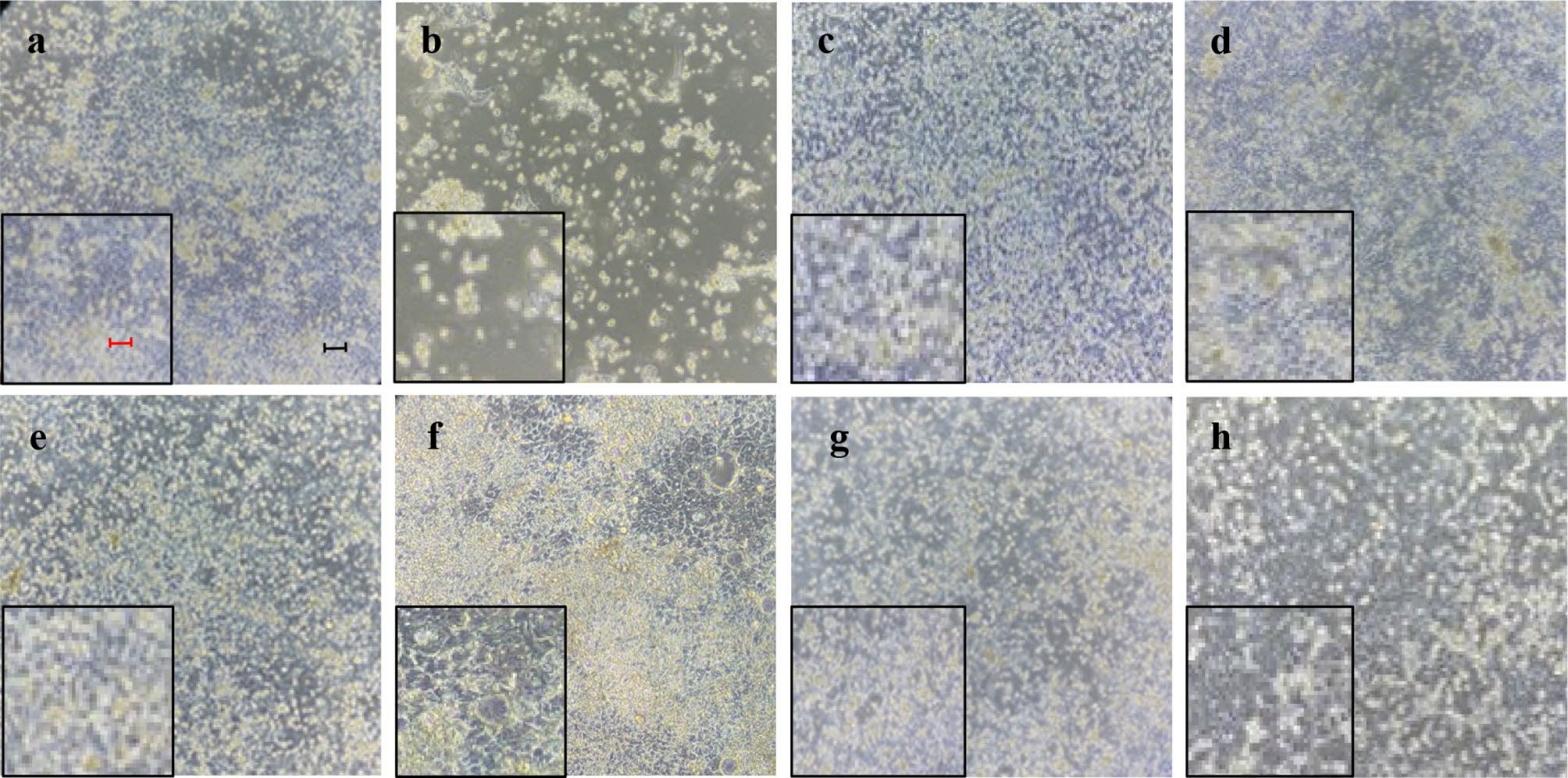
Microscopic observations of Caco-2 cell morphology after: (**a**) 72 h treatment with DMSO vehicle only; (**b**) 72 h pre-treatment with SA and subsequent with DMSO; (**c**, **d**) 72 h pre-treatment with SA and subsequent with 5 and 50 µM resveratrol respectively; (**e**, **f**) 72 h pre-treatment with SA and subsequent with 10 and 100 µM pterostilbene, respectively; (**g**, **h**) 72 h pre-treatment with SA and subsequent with 10 and 100 µM pterostilbene dimer, respectively. The morphology of cells treated with both SA and stilbenoids is similar to the condition of cells shown in (a), treated with DMSO vehicle only. It represents a homogeneous and compact cellular carpet very different from what is shown in (b). Every picture reports a 5 × magnification on the left for a better image evaluation. Black bar represents 10 µm, the red one, 50 µm.

After the pre-treatment with Sodium Arsenite (SA) and DMSO (SaDMSO) we found considerable variations of cell density and ability of adhesion to the substrate (Fig. 4a, b). Compared to the SaDMSO culture, the SaRV5 and SaRV50 cultures (see Table 5 for acronyms) had a comparable cell density slightly lower than DMSO. There was also less cellular stress and a greater ability to adhere to the substrate (Fig. 4c, d). Pterostilbene was effective in reversing arsenic damage (Fig. 4e, f), whereas (±)-trans-pterostilbene dehydrodimer hindered the stress caused by SA (Fig. 4g, h).

### Epigenomic studies

Considering the lack of information in the literature about epigenetic contribution of (±)-trans-pterostilbene dehydrodimer, (±)-*trans*-δ-viniferin and the information known about the other molecules object of this study, we wanted to take advantage from the preliminary data obtained about cellular morphology after treatments and in a particular way from the hypothetical cytotoxic effect of (±)-*trans*-δ-viniferin compared to the other molecules, to understand how this molecule behaves in an in vitro model at the epigenomic level. From treatments with resveratrol, pterostilbene and (±)-trans-pterostilbene dehydrodimer, no significant variation in the band pattern of Methylation-Sensitive Arbitrarily-Primed PCR (MeSAP-PCR) analyses was observed. Thus, no epigenomic action was observed both on Caco-2 and HepG-2 cell lines in relationship to the treatments with these previously-mentioned stilbenoids.

#### (±)-*trans*-δ-viniferin showed an epigenomic activity by changing genomic DNA methylation

**Figure 5:**
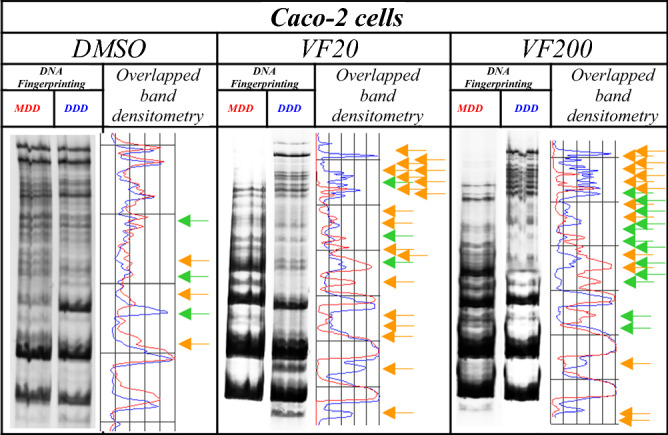
Representative MeSAP-DNA fingerprinting, and relative scanning densitometry, indicating genomic DNA methylation of Caco-2 cells treated with: (from left) DMSO – vehicle; 20 µM (±)-*trans*-δ-viniferin (VF20); 200 µM (±)-*trans*-δ-viniferin (VF200). Band pattern variation, in terms of intensification/weakening (green arrows) and appearance/disappearance (orange arrows) of bands was evaluated by densitometer scanning of mono-digested DNA (MDD, profile in red) in comparison with double-digested DNA (DDD, profile in blue). The number of total variations was reported in Table 1. Original blots/gels are presented in Supplementary Fig. 1.

**Figure 6:**
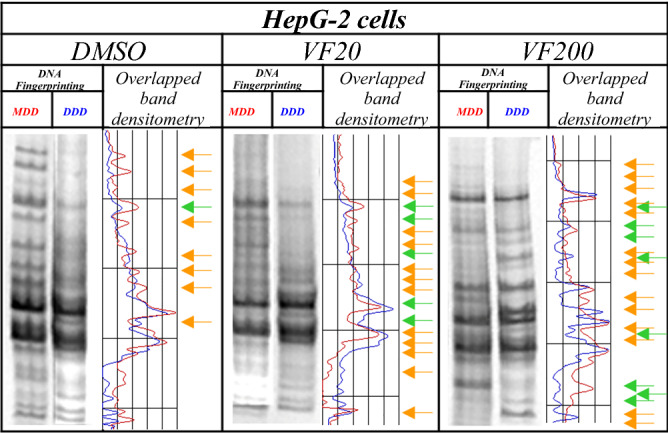
Representative MeSAP- DNA fingerprinting, and relative scanning densitometry, indicating genomic DNA methylation of HepG-2 cells treated with: (from left) DMSO–vehicle; 20 µM (±)-*trans*-δ-viniferin (VF20); 200 µM (±)-*trans*-δ-viniferin (VF200). Band pattern variation, in terms of intensification/weakening (green arrows) and appearance/disappearance (orange arrows) was evaluated by densitometer scanning of mono-digested DNA (MDD, profile in red) in comparison with double-digested DNA (DDD, profile in blue). The number of total variations was reported in Table 1. Original blots/gels are presented in Supplementary Fig. 1.

Interestingly, as is shown in Table 1 and Figs. 5, 6, treatment with (±)-*trans*-δ-viniferin caused significant changes in genomic DNA methylation of both Caco-2 and HepG-2 cells in a dose-dependent manner. Compared to cells treated with DMSO vehicle only used as control, in the (±)-*trans*-δ-viniferin-treated cells the band pattern variation was present with at least a double value. Moreover, these variations were minor than those obtained with 5-AzaC treatment. Indeed, treatment with B339H, a selective TET enzymes inhibitor, at two concentrations (B33 and B75), gave a different value of band pattern variation in the two cell lines. In particular, Caco-2 cells DNA resulted demethylated only by B75 treatment. On the contrary, HepG-2 cells DNA was mainly demethylated by B33 treatment, although the value relative to B75 treatment was surprisingly similar to those obtained with VF20 and VF200 treatments.

**Table 1:** Values of band pattern variation obtained by MeSAP analysis (± Standard Deviation of two independent experiments) and densitometer scanning of fingerprinting of mono-digested DNA (MDD) in comparison with double-digested DNA (DDD) expressing the demethylation of both treated cell lines DNA at genome level.

Cell line	Band pattern variation (± SD)					
	DMSO	VF20	VF200	5-AzaC	B33	B75
Caco-2	6 (0.10)	22°° (0.90)	26°° (1.26)	35°°° (1.40)	6 (0.23)	28°° (0.78)
HepG-2	9 (1.12)	17* (1.85)	22° (2.22)	25*** (2.11)	30°°° (2.87)	19** (2.00)

(*) 0.05 < *p* < 0.025; (**) 0.025 < *p* < 0.02; (***) 0.01 < *p* < 0.005; (°) 0.005 < *p* < 0.001; (°°) *p* < 0.001; (°°°) p <  < 0.001, calculated with the χ^2^ test in comparison with respective DMSO- treated cells value.

#### Treatment with stilbenoids is able to recover SA-induced DNA demethylation in Caco-2 cells

**Figure 7:**
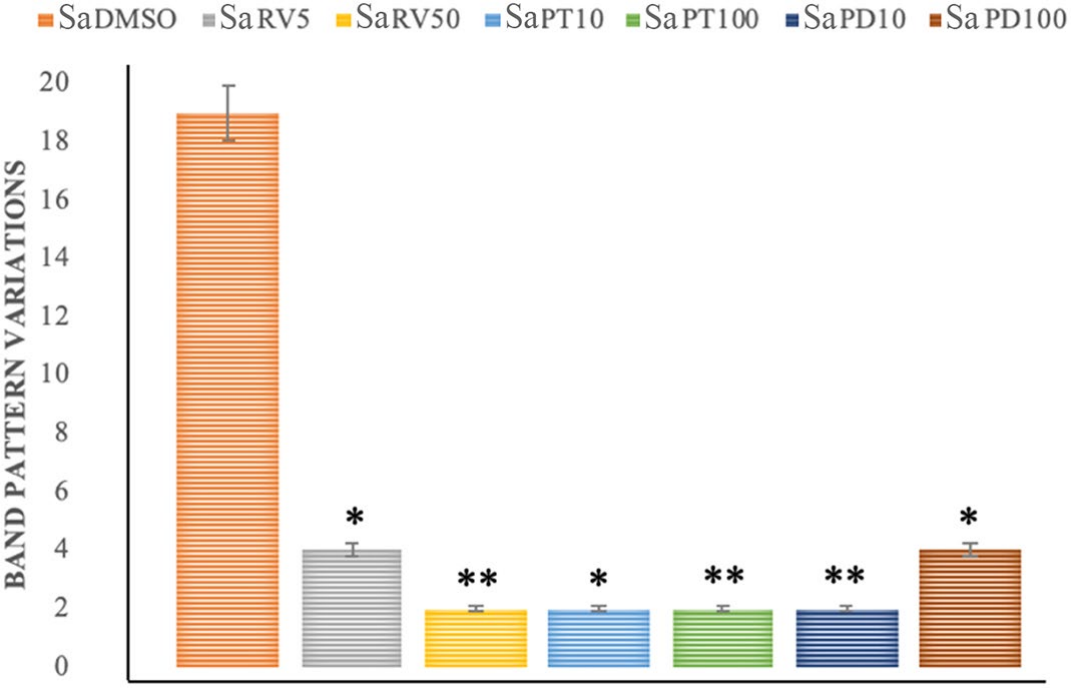
Representative values of band pattern variation obtained by MeSAP analysis (by two independent experiments) expressing the DNA genomic demethylation of Caco-2 cells pre-treated with SA and subsequently with each stilbenoid. (*) 0.01 < *p* < 0.005; (**) *p* <  < 0.001 calculated with the χ^2^ test in comparison with SA treatment.

Treatment with SA induced significant demethylation of genomic DNA, as demonstrated by MeSAP analysis (SaDMSO). In Table 2 and Fig. 7 the averages of two independent experiments of the combined treatments with a sequential addition of SA and each stilbenoid, were also reported. As it is possible to note, treatments with stilbenoids at a concentration 10-fold lower and similar to IC_50_ was able to significantly remedy SA-induced DNA demethylation.

**Table 2:** Values of band pattern variation obtained by MeSAP analysis (± Standard Deviation of two independent experiments) and densitometer scanning of fingerprinting of mono-digested DNA (MDD) in comparison with double-digested DNA (DDD) expressing the demethylation treated Caco-2 DNA at genome level.

Cell line	Band pattern variation (± SD)						
	SaDMSO	SaRV5	SaRV50	SaPT10	SaPT100	SaPD10	SaPD100
Caco-2	19 (0.95)	4* (0.20)	2** (0.10)	2** (0.10)	2** (0.10)	2** (0.10)	4* (0.20)

(*) 0.01 < *p* < 0.005; (**) *p* <  < 0.001 calculated with the χ^2^ test in comparison with respective DMSO- treated cells value.

### DNA-damage analysis

From treatments with resveratrol, pterostilbene and (±)-trans-pterostilbene dehydrodimer, no significant Olive Tail Moment (OTM) variation in Comet assay was observed. Thus, no clastogenic power was shown on both Caco-2 and HepG-2 cells by these stilbenoids.

#### (±)-*trans*-δ-viniferin showed a genotoxic power by inducing DNA strand breaks

**Figure 8:**
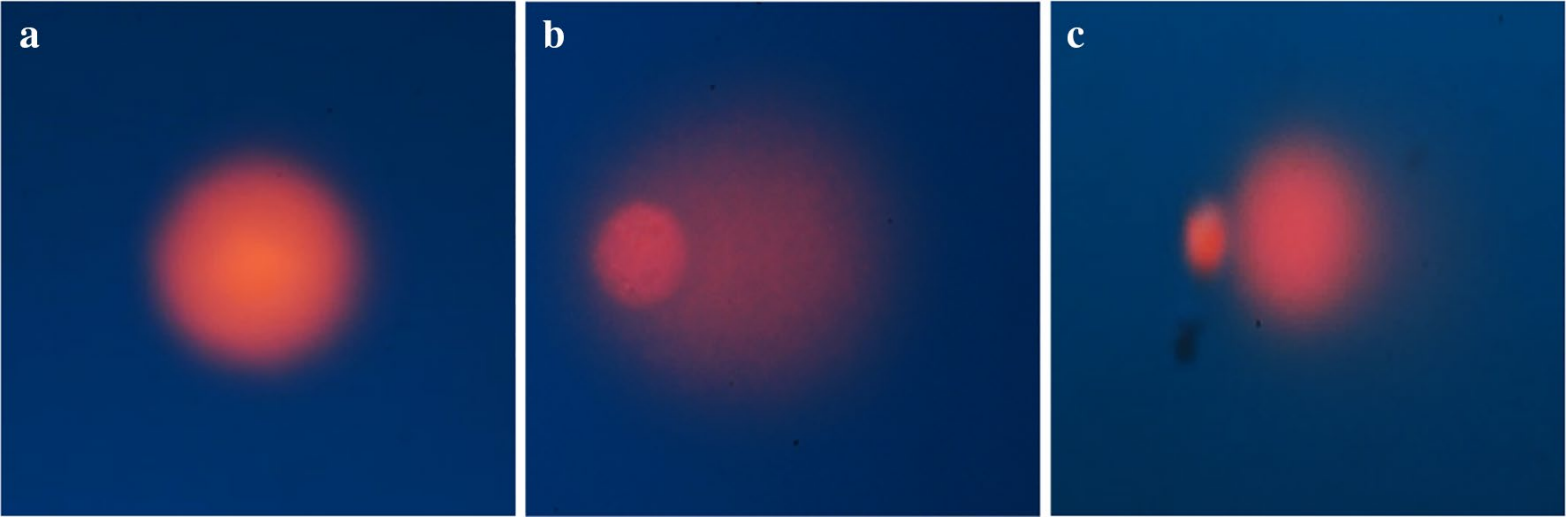
Representative pictures of alkaline comet assay (evidence of SSBs and DSBs): (**a**) DMSO—control Caco-2 cell undamaged nucleus; (**b**) VF200-treated Caco-2 comet; (**c**) VF200-treated HepG-2 comet. The principle of the assay is that under an electric field, fragmented DNA migrates out of the nucleoid body (also known as the "comet head") and forms a smeared DNA stain in the agarose gel (also known as the "comet tail"). Thus, the more is the comet tail (as more in **b** and less in **c**), the more DNA is fragmented, more DNA damage is present. Conversely, the more round is the nucleoid body (as in **a**), the less DNA is fragmented, less DNA damage is present. By CASP software (Biotools) it can be calculated the parameter “tail moment” to quantitatively express this concept.

Conversely, the analysis of OTM values by CASP software (Biotools) of the cells treated with (±)-*trans*-δ-viniferin, showed an increasing OTM mean values, in a dose-dependent regimen compared to those exposed to DMSO vehicle only (Table 3; Fig. 8). In particular, all the values referred to (±)-*trans* δ-viniferin treatments were significantly different from control, mainly for the HepG-2 cells treated at 200 µM concentration. Moreover, OTM averages from HepG-2 treated with B33, deviated considerably from the DMSO ones, and were instead close to the value of VF200. Conversely, for the aforementioned cell line, it is evident that the treatment with 5-AzaC, did not determine significant differences in the mean OTM values, compared to the control with DMSO.

**Table 3:** Values of OTM (averages) obtained by Comet assay (± Standard Deviation of two independent experiments) expressing the DNA damage inducing power of (±)-*trans*-δ-viniferin on both treated cell lines.

Cell line	Olive Tail Moment (OTM) (± SD)					
	DMSO	VF20	VF200	5-AzaC	B33	B75
Caco-2	6.65 (1.24)	10.91* (0.01)	12.73* (1.07)	15.22** (1.88)	8.22 (2.11)	20.55** (2.76)
HepG-2	2.02 (0.06)	6.51* (1.80)	22.41*** (3.61)	3.43 (0.48)	22.35*** (3,35)	23.05*** (4.01)

(*) 0.05 < *p* < 0.01; (**) 0.01 < *p* < 0.005; (***) *p* < 0.001, calculated with the χ^2^test in comparison with respective DMSO- treated cells value.

## Discussion

Stilbenoids, found in a variety of plant species, represent an important class of food bioactives. They are the most abundant antioxidants consumed by humans and have an important role in Mediterranean diet^17^. Although there is an increasing amount of specific literature data and several studies have generally ascertained their positive biological effects^18,19^, the ways by which they carry out their beneficial effects on human health have not been fully clarified. For this reason, we investigated the in vitro epigenetic modulatory activity of selected stilbenoids in differentiated Caco-2 cells and HepG-2 cells. In this regard, it should be remembered that differentiated Caco-2 cells are described in the literature as a normal polarized monolayer and therefore represent an in vitro model of intestinal cells^20^. HepG-2 cells, on the other hand, deriving from hepatocarcinoma, represent a cancerous model for specific nutrigenomic studies^21^. The stilbenoids here investigated were resveratrol, pterostilbene, (±)-*trans*-δ-viniferin and (±)-trans-pterostilbene dehydrodimer. Two cell lines were used for our studies: differentiated Caco-2, and HepG-2 cells.

Resveratrol, pterostilbene and (±)-trans-pterostilbene dehydrodimer, resulted to have no toxic effects, leaving the morphology of the treated cells unaltered. Interestingly, (±)-*trans*-δ-viniferin, a resveratrol dimer, induced changes in the cellular morphology resulting in a detached and rounded cells (Fig. 2, 3). The nutrigenomic approach and its informative potential were performed to clarify the cytotoxic effects showed by (±)-*trans*-δ-viniferin.

We also used compounds with a known demethylating effect such as 5-AzaC and B339H, as positive controls. It is known that 5-AzaC inhibits DNA methylation^22^ by a negative interaction with the methyl donor S-adenosine-methionine (SAM). B339H, a recently synthesized molecule, determines changes in DNA methylation by an indirect action of inhibition of TET enzymes, without inhibiting DNMT3A. In particular, a 33 μM treatment yields data relating to the inhibition of the TET1 enzyme alone and a 75 μM dose provides information about the inhibition of both enzymes^23^. Interestingly, the cell lines treated with these two molecules (culture acronym AZA, B33 and B75), appeared with morphological alterations. Similarly, cells treated with (±)-*trans*-δ-viniferin (Figs. 2 and 3) showed morphological alterations. Prompted by this analogous behavior, we evaluated the epigenetic/epigenomic potential of (±)-*trans*-δ-viniferin and we found that it significantly modified the DNA methylation of the cells (Table 1, Figs. 5 and 6). In fact, in Caco-2 and HepG-2 cell lines variations of the band pattern were found significantly different in comparison with the cells with DMSO vehicle. Remarkably, these values were very similar to those obtained, in both cell lines, with 5-AzaC, suggesting that (±)-*trans*-δ-viniferin most likely increases DNA demethylation via SAM-DNMTs interaction. In addition, by treating cells with B339H, which promotes TET enzymes inhibition, we found a sustained genomic DNA demethylation with different behavior in Caco-2 and HepG-2 cells. At the concentration inhibiting both the TET enzymes (75 μM), Caco-2 cells showed a high increase of DNA demethylation while HepG-2 showed the same behavior at both the doses with a greater effect at 33 μM concentration, which should give the inhibition of TET1 enzyme only. Since treatments with 5-AzaC, (±)-*trans*-δ-viniferin and B339H showed similar effect, we can affirm that (±)-*trans*-δ-viniferin acts as a demethylating agent. An issue remains open: the increase in demethylation of genomic DNA in the presence of B339H that inhibits TET enzymes. Further studies will need to be carried out to clarify these intriguing characteristics. B339H is a molecule that has only recently been put on the market, consequently there are very few published studies in which it has been used. We also investigated the ability of selected stilbenoids to counteract the effects of a frequent and unavoidable epigenetic insult for humans, like arsenic, a non-metal with high environmental pervasiveness, introduced daily through drinking water in micromolar concentrations, whose mode of action is still unclear^24^. In order to show what might happen in a normal intestinal cell model in vitro and offer a projection of what likely occurs in vivo, we submitted Caco-2 cells to a combined treatment (a pre-treatment with SA, followed by a treatment with stilbenoids). To the best of our knowledge, this is the first study of such a combined treatment. As expected, the treatment with SA caused remarkable alterations in the cell morphology, with small size and not adherent cells (Fig. 4b). Interestingly, in the subsequent addition of stilbenoids (Fig. 4c–h), reversed in a relevant percentage, the morphological damage induced by SA insult, so that the cells reverted the phenotype like control ones. In addition, more interesting are the results regarding DNA methylation, also gained by the combined treatments (Fig. 7). According to the literature^25^, pre-treatment of Caco-2 cells with SA induces DNA demethylation. When pre-treatment with arsenic is followed by treatment with stilbenoids, using concentrations similar to those they would have in the intestinal lumen, the global methylation levels of DNA was similar or even slightly lower than the control ones (Table 2).

The atypical behavior of (±)-*trans*-δ-viniferin in regard of DNA methylation, prompted us to investigate whether this stilbenoid is also able to induce DNA damage, since the relationship between chromatin modifications and double-strand break signaling and repair is well known^26^. By carrying out alkaline comet test, we highlighted that (±)-*trans*-δ-viniferin showed a dose-dependent cytogenotoxic effect at both concentrations in both the cell lines (Table 3; Fig. 8) with a peculiar difference for the HepG-2 cells for which the OTM variation induced in VF200 is significantly greater compared to the control with DMSO (Fig. 8c).

Interesting data, useful to better understand (±)-*trans*-δ-viniferin action, came from the comet test applied to cells treated with positive-control molecules whose action is specific but different on DNA methylation. For example, 5-AzaC, resulted cytogenotoxic for differentiated Caco-2 cells but no for HepG-2 cells. Moreover, B339H induces a strong and significant cytogenotoxicity in HepG-2 cells but it was able to induce DNA damage in Caco-2 cells only at 75 μM. By looking for behavior analogies with (±)-*trans*-δ-viniferin comet data, it can be suggested that this stilbenoid can exert its effect by modulating DNA methylation preferentially by TETs rather than by DNMTs.

In conclusion, by applying the nutrigenomic approach, we investigated the in vitro epi-genotoxic properties of resveratrol, pterostilbene and their dimers (±)-*trans*-δ-viniferin and (±)-trans-pterostilbene dehydrodimer. In particular, we demonstrated that resveratrol, pterostilbene and (±)-trans-pterostilbene dehydrodimer, did not modulate genomic DNA methylation of normal and cancer cells, and that they were able to revert morphologic damage and genomic DNA demethylation when added following a treatment with arsenic in concentration similar to that can be assumed by drinking water. The latter topic could represent a great help to the world of animal husbandry and irrigation-dependent crop production. Thus, we can assert that resveratrol, pterostilbene and its dimer, already described as activators of DNA repair mechanisms in various cancer cell lines, including prostate, colon and breast cancer cells^8,27,28^ even showed, as demonstrated for the first time by our data, a great reversal power of arsenic-induced DNA demethylation. Considering that, we have tested these molecules, also contained in Mediterranean diet, in an in vitro model that mimics in vivo conditions, our data give the possibility to extrapolate the suggestive working hypothesis that some of these molecules can be used in the bio-fortification of normal foods to obtain a diet with a second level of health effect.

Among the studied stilbenoids, (±)-*trans*-δ-viniferin showed epigenotoxic and cyto-genotoxic effects on Caco-2 and HepG-2 cell lines at 200 μM concentration, with a mechanism of action that seems to favor the involvement of intracellular DNA methylation machinery, surely, starting further studies mainly addressed to see (±)-*trans*-δ-viniferin as a chemopreventive agent.

## Methods

### Cell models

All the cell lines were cultured as previously described^29–31^.

#### Caco-2

Caco-2 (ATCC, Palo Alto, CA, USA) represent a human colon epithelial cancer cell line used as a study model for the mechanisms underlying colon cancer development, toxicology and, above all, for the analysis of colon cancer processes, absorption and metabolism in food science, nutrition and drug discovery^32^.

Caco-2 cells, cultured as a monolayer, differentiate after 21 days and are a model for studying the paracellular movement of compounds across a monolayer. Furthermore, they express several morphological and biochemical characteristics typically shown by mature small intestinal normal enterocytes^33,34^. Numerous studies have revealed that differentiated Caco-2 cells constitute a polarized monolayer characterized by domes, with microvilli on the apical side and tight junctions between adjacent cells^20^. Thus, Caco-2 cells were allowed to grow to confluence and differentiated after 21 days for here-showed biological experiments.

#### HepG-2

The HepG-2 cell line (ATCC, Palo Alto, CA, USA), a hepatic cell line derived from a human hepatoblastoma, expresses a variety of liver-specific metabolic functions^35^. These cells have an epithelial-like morphology and secrete a variety of important plasma proteins. HepG-2 cells are a suitable model for studying the metabolic fate/effects of xenobiotic on metabolism.

### Molecules for pre-treatments, treatments and positive controls

Resveratrol and pterostilbene are commercially available (Merck Life Science, Milano, Italy); dimers (±)-*trans*-δ-viniferin and (±)-trans-pterostilbene dehydrodimer (Fig. 1) were synthesized according to reported procedures^36^.

For treatments with (±)-*trans*-δ-viniferin and positive control molecules, we used both cell lines while for the combined treatments we used the Caco-2 cell line only. The determination of treatment times was established on a functional basis. Differentiated Caco-2 cells were treated for 3 h mimicking the average time of in vivo intestinal absorption. In parallel, for HepG-2 liver cancer cells, a time of 24 h was used simulating in vivo hepatic metabolism.

Bobcat339 hydrochloride (B339H), a selective inhibitor of TET1 and TET2 (at doses 33 and 75 μM, respectively), that does not inhibit DNMT3A was selected for DNA methylation studies^37^. The treatment was carried out for 72 h in order to evidence appreciable DNA methylation differences, after, at least, two or three cell cycles.

Moreover, as second DNA methylation-specific positive control, 5-Azacytidine (5-AzaC, 10 μM), a widely known molecule that acts preferentially on the complex DNMTs-S-adenosine methionine (SAM), was used for 48 h (with fresh adding at 24 h due to low half-life of this molecule).

For B339H and 5-AzaC concentration, literature data were used^37,38^.

To simulate a specific genotoxic insult, we pretreated the Caco-2 cells with sodium arsenite (SA) [10 µg/L] for 72 h and then incubated the cells with each stilbenoid for 3 h. This concentration of arsenic was used in accordance with current legislation in Europe and United States and according to the guidelines of the World Health Organization that defines a limit value of arsenic in drinking water^39^.

All stilbenoids and B339H were solubilized in dimethyl sulfoxide (DMSO) taking care that the vehicle volume is always less than 0.01% of the cell medium to avoid self-effect on the followed biological and epigenetic/molecular endpoints^40,41^. SA and 5-AzaC were dissolved in cell medium. With the aim to mimic the low concentrations occurring in the intestinal lumen after ingestion of foods or vegetables rich in these substances, we have chosen to treat the cells with two doses of stilbenoids, one corresponding to the respective IC_50_ concentration and the other one order of magnitude lower than the IC_50_.

#### IC_50_ calculation

For the treatments of Caco-2 cells with resveratrol (indicated as stilbenoid **1** in Fig. 1), we used one of the concentrations reported and used by Storniolo et al.^42^. HepG-2 cell line was treated with resveratrol at the same concentration used for Caco-2 cells, referring to the concentrations used by Akashina et al. and Aja et al.^43,44^.

**Figure 9:**
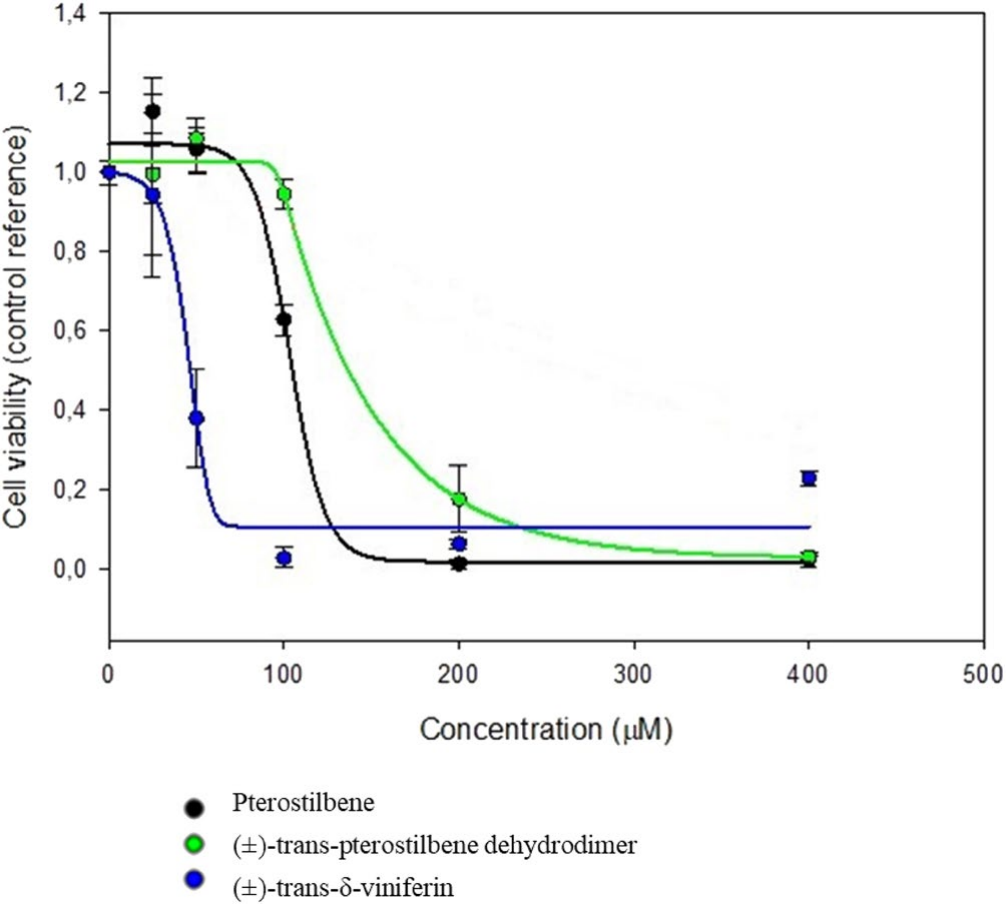
Caco-2 cell lines IC_50_ curve for stilbenoids after 24 h of stimulation.

In order to evaluate the IC_50_ (Fig. 9), Caco-2 cell line was treated with stilbenoids (indicated as **2**–**4** in Fig. 1) for 24 h (Table 4).

**Table 4:** Stilbenoids IC_50_ on Caco-2 cell lines.

Stilbenoid	IC_50_ (µM)
Pterostilbene	100
(±)-trans-pterostilbene dehydrodimer	100
(±)-*trans*-δ-viniferin	200

### Cell-culture planning

All the cell-cultures, pre-treatments and treatments are summarized in Table 5. A total amount of 19 cell-cultures was carried out, 7 of which were also pre-treated.

**Table 5:** Cell cultures/treatments planning.

Pre-treatment (µg/L)	Treatment (µM)	Cell-culture acronym	Cell line(s)*	Incubation time (h)
–	DMSO	DMSO	C	3
–	Resveratrol (5)	RV5	C	3
–	Resveratrol (50)	RV50	C	3
–	Pterostilbene (10)	PT10	C	3
–	Pterostilbene (100)	PT100	C	3
–	(±)-trans-pterostilbene dehydrodimer (10)	PD10	C	3
–	(±)-trans-pterostilbene dehydrodimer (100)	PD100	C	3
–	(±)-*trans*-δ-viniferin (20)	VF20	C, H	3, 24
–	(±)-*trans*-δ-viniferin (200)	VF200	C, H	3, 24
–	B339H (33)	B33	C, H	72
–	B339H (75)	B75	C, H	72
–	5-AzaC (100)	AZA	C, H	24 + 24
SA [10]	DMSO	SaDMSO	C	72 + 3
SA [10]	Resveratrol (5)	SaRV5	C	72 + 3
SA [10]	Resveratrol (50)	SaRV50	C	72 + 3
SA [10]	Pterostilbene (10)	SaPT10	C	72 + 3
SA [10]	Pterostilbene (100)	SaPT100	C	72 + 3
SA [10]	(±)-trans-pterostilbene dehydrodimer (10)	SaPD10	C	72 + 3
SA [10]	(±)-trans-pterostilbene dehydrodimer (100)	SaPD100	C	72 + 3

*C* Caco-2 cells, *H* HepG-2 cells.

### Morphological observations

Morphological cell aspects were considered to evaluate the onset of changes induced by treatments. By respecting the same microscope magnification (Leica, 40 ×), the observations were aimed to evaluate: (i) cell density, (ii) adhesion to the substrate and (iii) presence of cell suffering (bubble and/or shriveled cells). With these bases, cellular carpets after treatments were photographed and compared with the control ones.

### Genomic DNA isolation

Isolation of genomic DNA from cells was carried out with the PureLink Genomic DNA Kit (Invitrogen, UK) as previously described^45^ and DNAzol (Invitrogen®) following the manufacturer recommendations. The obtained DNA was quantified by NanoDrop® ND-1000.

### Epigenomic assessment of DNA methylation

To assess the possible genomewide changes in DNA methylation, Methylation-Sensitive Arbitrarily-Primed PCR (MeSAP-PCR) was performed as previously described^25^ on cells treated with stilbenoids and DMSO vehicle only.

### Quantitation of DNA damage by Comet Assay

Comet Assay method^46^, also called Single Cell Gel Electrophoresis (SCGE), is a micro-electrophoretic technique through which the nuclear DNA, possibly fragmented, can be visualized by observing the shape of the nuclei and the presence of a tail similar to a comet visible through observation under a epifluorescence microscopy. The extent of DNA damage is visually assessed by visualization of the comet's tail (see Fig. 8b and c); an image analysis software is also available to measure different parameters related to the dimensions of the comet's tail. With alkaline Comet assay, it is possible to detect DNA damage of different entities: single and double DNA strand breaks, presence of adducts, alterations convertible into breaks such as labile alkali sites or incisions caused by the repair mechanisms by excision of nucleotides. We performed alkaline Comet assay as previously described^45,47^ with some differences. Briefly, at the end of the incubation times of the different treatments, the treated and untreated cells were recovered to be analyzed using the OxiSelect™ Comet Assay kit (Cell Biolabs Inc, San Diego, CA), a rapid and sensitive kit for measuring DNA cellular damage. Once recovered, the cells were mixed with molten agarose and then placed on the slide. After treating the slides with a lysis and alkaline solution the samples on the slide were subjected to horizontal electrophoresis, in order to separate the intact DNA from the damaged DNA fragments. After electrophoresis, the samples were air dried, stained with a fluorescent DNA probe and visualized by epifluorescence microscopy (Nikon). Lastly, the images were analyzed with CASP software (Biotools) to obtain the “tail moment”, a quantitative parameter that express the length of the tail in function of the nucleus size.

## Supplementary Information

Supplementary Information.

## Data Availability

The data contained in this manuscript has been obtained from scientific experiments. They have been elaborated and cross-linked in order to obtain information useful to assert some hypotheses and show some results on topic. Raw data are always available if requested from the corresponding author by subjects who are entitled to request them and who provide valid reasons. Conversely, to avoid plagiarism and fraudulent publications they will be denied.
