# Supplementary material for: Nutritional epigenomic and DNA-damage modulation effect of natural stilbenoids
Source: Sci Rep. 2023 Jan 12;13:658. doi: 10.1038/s41598-022-27260-1 (PMC9837110; doi:10.1038/s41598-022-27260-1)
Supplement: Supplementary file 1 — Supplementary Information. [file 41598_2022_27260_MOESM1_ESM.docx]

**Supplementary files**


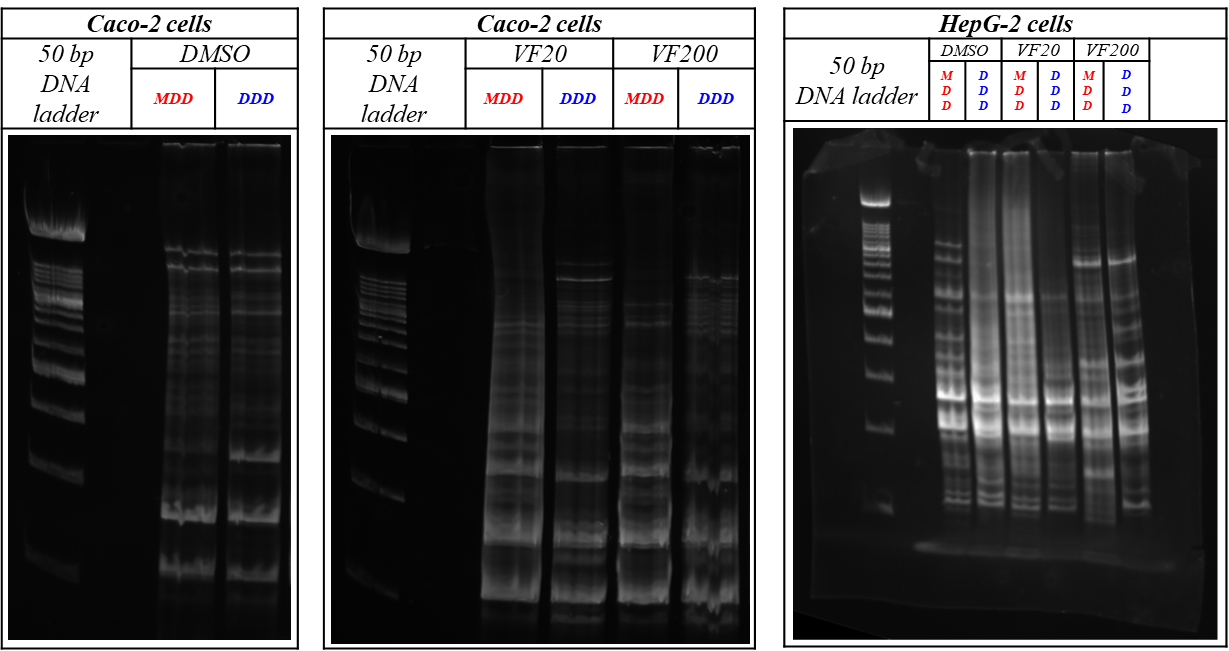


Supplementary figure 1 – Unprocessed original full-length acrylamide/polyacrylamide gels, chosen and inverted black on white for figures 5 and 6. Caco-2 cells (left) treated with DMSO – vehicle; Caco-2 cells (center) treated with 20 or 200 µM (±)-trans δ-viniferin; HepG-2 cells (right) treated with DMSO – vehicle, 20 or 200 µM (±)-trans δ-viniferin.
